# Renal vasoconstriction is augmented during exercise in patients with peripheral arterial disease

**DOI:** 10.1002/phy2.154

**Published:** 2013-11-07

**Authors:** Rachel C Drew, Matthew D Muller, Cheryl A Blaha, Jessica L Mast, Matthew J Heffernan, Lauren E Estep, Jian Cui, Amy B Reed, Lawrence I Sinoway

**Affiliations:** Penn State Hershey Heart and Vascular Institute, Penn State University College of MedicineHershey, Pennsylvania

**Keywords:** Exercise, oxidative stress, peripheral arterial disease, renal

## Abstract

Peripheral arterial disease (PAD) patients have augmented blood pressure increases during exercise, heightening their cardiovascular risk. However, it is unknown whether patients have exaggerated renal vasoconstriction during exercise and if oxidative stress contributes to this response. Eleven PAD patients and 10 controls (CON) performed 4-min mild, rhythmic, plantar flexion exercise of increasing intensity (0.5–2 kg) with each leg (most and least affected in PAD). Eight patients also exercised with their most affected leg during ascorbic acid (AA) infusion. Renal blood flow velocity (RBFV; Doppler ultrasound), mean arterial blood pressure (MAP; Finometer), and heart rate (HR; electrocardiogram [ECG]) were measured. Renal vascular resistance (RVR), an index of renal vasoconstriction, was calculated as MAP/RBFV. Baseline RVR and MAP were similar while HR was higher in PAD than CON (2.08 ± 0.23 vs. 1.87 ± 0.20 au, 94 ± 3 vs. 93 ± 3 mmHg, and 72 ± 3 vs. 59 ± 3 bpm [*P* < 0.05] for PAD and CON, respectively). PAD had greater RVR increases during exercise than CON, specifically during the first minute (PAD most: 26 ± 5% and PAD least: 17 ± 5% vs. CON: 3 ± 3%; *P* < 0.05). AA did not alter baseline RVR, MAP, or HR. AA attenuated the augmented RVR increase in PAD during the first minute of exercise (PAD most: 33 ± 4% vs. PAD most with AA: 21 ± 4%; *P* < 0.05). In conclusion, these findings suggest that PAD patients have augmented renal vasoconstriction during exercise, with oxidative stress contributing to this response.

## Introduction

Peripheral arterial disease (PAD) is a cardiovascular disease that affects ∼10% of people worldwide and 15–20% of those over 70 years of age (Peach et al. 2012). Risk factors for PAD include smoking, hypertension, diabetes and hyperlipidemia, and patients with PAD have a significantly higher risk of dying from cardiovascular disease than those without PAD. This disease is characterized by narrowing of blood vessels due to atherosclerosis, resulting in a reduced ankle-brachial index (ABI), for example, <0.9. A lower ABI correlates with more severe disease, as well as an increased risk for cardiovascular events (Belch et al. 2003). The main symptom of PAD is intermittent claudication, consisting of limb pain or ache during physical activity that is relieved by rest. PAD can often be asymptomatic, leaving many people with PAD unaware of their condition but still at elevated risk of atherothrombotic events such as myocardial infarction or stroke (Hirsch et al. 2001).

Exercise causes redistribution of blood away from “inactive” vascular beds such as the kidney towards working skeletal muscle, as well as increases in blood pressure, heart rate (HR), and ventilation in response to feedforward (central command) and feedback (muscle afferents) mechanisms (Raven et al. 2006). As the kidneys receive ∼25% of resting cardiac output (Zelis et al. 1988), renal vasoconstriction during exercise is an important reflex mechanism involved in maintaining an appropriate blood pressure depending on the exercise intensity. Muscle mechanoreflex and metaboreflex activation, due to stimulation of group III and IV muscle afferent nerve fibers by mechanical distortion of muscle fibers and metabolic by-products within active skeletal muscle, respectively, can both influence the level of renal vasoconstriction that occurs during exercise in humans (Middlekauff et al. 1997; Momen et al. 2003). The muscle mechanoreflex in particular appears to play a predominant role in this reflex response in both humans (Momen et al. 2003) and animals (Koba et al. 2006). Renal vasoconstriction during exercise is augmented in heart failure patients (Middlekauff et al. 2000), primarily due to exaggerated muscle mechanoreflex activation (Middlekauff et al. 2001), which has also been demonstrated in animal models of PAD (McCord et al. 2011; Lu et al. 2013), heart failure (Koba et al. 2008; Morales et al. 2012), and hypertension (Mizuno et al. 2011). In agreement with previous studies (Lorentsen 1972; Baccelli et al. 1999; Bakke et al. 2007), our group has recently shown that PAD patients have augmented blood pressure increases during lower-limb exercise (Muller et al. 2012). However, it is currently unknown whether greater renal vasoconstriction occurs during exercise in PAD patients.

Additionally, this augmented blood pressure increase in PAD patients was attenuated during infusion of the antioxidant ascorbic acid (AA; vitamin C) (Muller et al. 2012). Production of oxidants such as reactive oxygen species is increased by many of the risk factors for atherosclerosis (Cai and Harrison 2000). This oxidative stress causes endothelial dysfunction and atherosclerotic plaque formation that in itself leads to turbulent blood flow in the narrowed vessels, which further exacerbates oxidant production (Harrison et al. 2003). PAD patients have higher markers of oxidative stress (Mueller et al. 2004; Loffredo et al. 2007), as well as lower AA levels than controls (Langlois et al. 2001). Oxidative stress has been shown to augment the blood pressure and renal sympathetic nerve activity increases to static muscular contraction in a rat model of heart failure, likely due to sensitization of muscle mechanoreceptors (Koba et al. 2009). Therefore, we hypothesized that first, the exercise-induced increase in renal vasoconstriction would be augmented in PAD patients. Second, we hypothesized that this augmented renal vasoconstrictor response in PAD patients could be attenuated with AA, suggesting that oxidative stress contributes to this exaggerated response.

## Methods

### Ethical approval

The experimental protocol was approved by the Institutional Review Board of the Penn State Milton S. Hershey Medical Center and conformed to the Declaration of Helsinki. The studies were performed in the Clinical Research Center of the Penn State Milton S. Hershey Medical Center. The purpose and risks involved were explained to all subjects before written informed consent was obtained.

### Subjects

Recruitment involved screening 2302 PAD patients in the Heart and Vascular Institute at the Penn State Milton S. Hershey Medical Center. Inclusion criteria included being <75 years of age, body mass index of <35 kg m^2^, ABI <0.9 and/or symptomatic with intermittent claudication, and not having renal disease, coronary artery disease, heart failure, diabetes, chronic obstructive pulmonary disease, or peripheral neuropathy. Around 95% of the patients were excluded due to being >75 years of age (*n* = 665), not currently experiencing claudication (*n* = 586), renal disease (*n* = 190), coronary artery disease or previous myocardial infarction (*n* = 480), heart failure (*n* = 154), diabetes (*n* = 563), chronic obstructive pulmonary disease (*n* = 181), and/or amputation or wounds (*n* = 522). Of the 60 patients who met inclusion criteria, 14 volunteered to participate in the study. Renal blood flow velocity (RBFV) could not be measured in two patients and one patient developed premature ventricular contractions during exercise (>10 min^−1^). Therefore, data on 11 patients (eight men, three women) with Fontaine Stage I-II PAD are described here. These patients’ medications included β-blockers (*n* = 3), statins (*n* = 7), antihypertensives (*n* = 7), and non-steroidal anti-inflammatory drugs (*n* = 9). Antihypertensive medications, including angiotensin-converting enzyme inhibitors and β-blockers, were withheld on the morning of the study visit and were taken at the end of the visit. This meant there was a 12- or 24-h withdrawal period (depending on the drug), which allowed some time for clearance of the medications, although we cannot be certain that this period was sufficient. One patient was a current smoker and five patients were former smokers. It should be noted that mean arterial blood pressure (MAP) and HR data from all 11 PAD patients in this study were included in our previous study (Muller et al. 2012). The study by Muller et al. included MAP and HR data from 13 PAD patients, so the MAP and HR data in these two studies are from most of the same individuals. However, the number of patients in each group is slightly different, which explains why there are slight differences in the mean data of these groups in these two studies. Therefore, we have included MAP and HR data from 11 patients in this study as RBFV could be measured in these individuals.

In addition, 10 age-matched, healthy controls (CON; seven men, three women) were recruited to participate in the study. CON subjects were normotensive, had no history of cardiovascular disease, were not taking any current medications, and were in good health. It should be noted that MAP and HR data from 7 of the 10 CON subjects in this study were included in our previous study (Muller et al. 2012). RBFV could be measured in these seven individuals and we recruited a further three CON subjects in whom RBFV could be measured. Therefore, the MAP and HR data from the 10 CON subjects in this study and the nine CON subjects in the study by Muller et al. are from most of the same individuals. However, the number of subjects in each group is slightly different, which explains why there are slight differences in the mean data of these groups in these two studies. All subjects were asked to refrain from performing strenuous exercise, ingesting caffeine and alcohol for 24 h, and ingesting food for 8 h prior to their study visits.

### Experimental protocol

Subjects lay in a supine position and had their ABIs measured on both their left and right sides before performing the exercise trials. Subjects then had one of their feet strapped onto a footplate attached to a pulley system, which was designed so increasing weights could be added to incrementally increase the exercise intensity when performing plantar flexion. After subjects were settled for at least 10 min, 3 min of baseline was followed by one-legged, rhythmic plantar flexion for 4 min. Subjects were instructed to point their toes repeatedly in time to a metronome set at a rate of 30 contractions min^−1^. The weight applied in the pulley system during the first minute of exercise was 0.5 kg and an additional 0.5 kg was added every minute so by the fourth minute of exercise, 2 kg of weight was applied. Subjects were asked to rate their perceived level of exertion on a 6–20 scale (Borg 1982) and pain on a 0–10 scale 45 sec into each workload. After the end of exercise, there was a 2-min recovery period. After a 20-min rest period, the same exercise was performed with the opposite leg. PAD patients performed exercise with their most affected leg first (PAD-most), allowing for measurement of their maximal response first, followed by their least affected leg (PAD-least). CON subjects performed exercise with their right and left legs in random order, as none of these subjects had disease in either leg.

A subset of eight PAD patients returned for a second visit where they performed the same exercise trial as in their first visit with their most affected leg during AA infusion. The duration between patients’ first and second visits was usually 1–2 weeks. Patients were instrumented the same as previously and after patients were settled for at least 10 min, a 3-min pre-infusion baseline occurred. Intravenous AA (Bioniche Pharma, Lake Forest, IL) was then given via a loading dose of 45 mg kg^−1^ in 100 mL of saline over 20 min, followed by a maintenance dose of 15 mg kg^−1^ in 33 mL of saline for the remainder of the visit (with concentrations based on 160 mL of saline). High-dose AA infusion has been shown to increase plasma AA levels to >15 times normal physiological levels (Monahan et al. 2004). Once the loading dose was administered, patients performed the same plantar flexion exercise as previously with their most affected leg (PAD-most with AA).

### Cardiovascular and experimental measurements

The R-R interval was measured using a three-lead electrocardiogram (ECG, Cardiocap/5; GE Healthcare, Waukesha, WI), from which HR was derived. MAP was measured beat-to-beat using a finger cuff (Finometer; FMS, Arnhem, Netherlands) and was calibrated to baseline MAP measurements taken in triplicate before each trial using an arm cuff (Philips SureSigns VS3, Andover, MA). Respiratory movements were measured using a pneumography belt placed around the abdomen. Force produced during contraction was measured using a load cell on the pulley system and movement of the footplate using a potentiometer. An analog-to-digital converter sampled data at 200 Hz and data was displayed and recorded for off-line analysis (PowerLab; ADInstruments, Castle Hill, NSW, Australia).

RBFV was measured using Doppler ultrasound (HDI 5000; ATL Ultrasound, Bothell, WA) via the anterior abdominal approach, as described previously (Momen et al. 2003; Wilson et al. 2007). The renal artery was scanned using a 2–5 MHz curved-array transducer with a 2.5 MHz pulsed Doppler frequency. The insonation angle of the probe to the artery was ≤60° and the focal zone was set at the depth of the renal artery. The transducer was held in the same place for each trial and data were obtained during the same phase of the respiratory cycle within each subject.

### Data and statistical analyses

Raw data files were analyzed to produce beat-to-beat values for HR and MAP. Renal vascular resistance (RVR), an index of renal vasoconstriction, was calculated as MAP/RBFV (Momen et al. 2003; Wilson et al. 2007). Although our group has published MAP responses to exercise with and without AA in PAD patients and CON subjects previously (Muller et al. 2012), MAP data are included in this study as MAP is used to calculate RVR. Both MAP and RBFV are presented here, so it can be observed whether changes in RVR were due to changes in MAP, RBFV, or both of these variables. As we have also published HR responses to exercise with and without AA in PAD patients and CON subjects previously (Muller et al. 2012), HR data are included in this study to present a more comprehensive picture of the systemic responses to exercise in PAD and CON in the context of this study focusing on renal vascular responses to exercise in these groups. Group means were calculated for each phase and each trial. Means were calculated for each minute of exercise, corresponding to the increasing intensity. All values are expressed as mean ± SEM. Baseline differences were identified using independent-samples *t*-tests. Differences in cardiovascular responses were identified using repeated measures analysis of variance and independent- and paired-samples *t*-tests. Differences in ratings of perceived exertion (RPE) and pain were identified using independent-samples Mann–Whitney *U* tests and paired-samples Wilcoxon signed rank tests. Statistical significance was set at *P* < 0.05 and all statistical analysis was performed using SPSS (IBM, Armonk, NY).

## Results

It should be noted that most of the PAD patients’ and CON subjects’ characteristics, baseline values, MAP and HR responses to exercise with and without AA, and RPE and pain scores during exercise with and without AA presented in this study are similar to those values reported in our previous study (Muller et al. 2012). This is due to the PAD and CON groups in both studies including most of the same individuals (see Subjects section in Methods). However, as the number of individuals in these groups is slightly different between the two studies, these data are reported here in support of the novel RVR and RBFV data illustrating responses to exercise with and without AA in PAD patients and CON subjects.

PAD patients and CON subjects’ characteristics and baseline values are shown in Table 1. PAD and CON groups were of similar age, height, weight, body mass index, systolic blood pressure, diastolic blood pressure, MAP, RBFV, and RVR, but HR was higher in PAD than in CON (*P* < 0.05). PAD had significantly lower ABIs in both the most and least affected legs compared with CON (*P* < 0.05).

**Table 1:** Characteristics and baseline values for PAD patients and age-matched, healthy controls

	PAD	CON
Number of subjects (men/women)	11 (8/3)	10 (7/3)
Age (years)	65 ± 2	65 ± 2
Height (m)	1.69 ± 0.03	1.74 ± 0.03
Weight (kg)	79 ± 4	78 ± 4
BMI (kg m^−2^)	27.7 ± 1.2	25.8 ± 0.7
SBP (mmHg)	137 ± 5	127 ± 5
DBP (mmHg)	77 ± 2	77 ± 2
MAP (mmHg)	94 ± 3	93 ± 3
HR (bpm)	72 ± 3*	59 ± 3
RBFV (cm sec^−1^)	51.4 ± 6.2	55.1 ± 6.2
RVR (au)	2.1 ± 0.2	1.9 ± 0.2
ABI (most affected leg)	0.62 ± 0.06*	1.04 ± 0.03
ABI (least affected leg)	0.81 ± 0.05*	1.13 ± 0.04

Note that data for all variables except RBFV and RVR are similar to data published in our previous study (see Subjects section in Methods; Muller et al. 2012). PAD, peripheral arterial disease; CON, controls; BMI, body mass index; SBP, systolic blood pressure; DBP, diastolic blood pressure; MAP, mean arterial blood pressure; HR, heart rate; RBFV, renal blood flow velocity; RVR, renal vascular resistance; ABI, ankle-brachial index.

* Significantly different from CON (*P* < 0.05).

**Figure 1:**
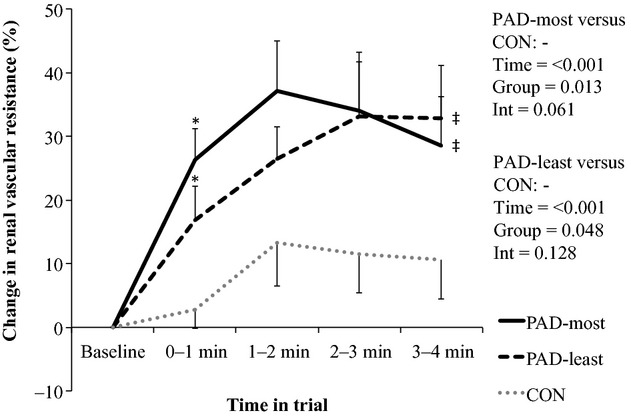
Percent change from baseline in renal vascular resistance for peripheral arterial disease (PAD) patients and age-matched, healthy controls (CON) during 4 min of mild, rhythmic, plantar flexion exercise of increasing intensity (0.5–2 kg) with each leg (most and least affected legs for PAD; first leg for CON). Int, interaction. *Significantly different from CON at specific time point (*P* < 0.05). ^‡^Significantly different from CON (*P* < 0.05).

**Figure 2:**
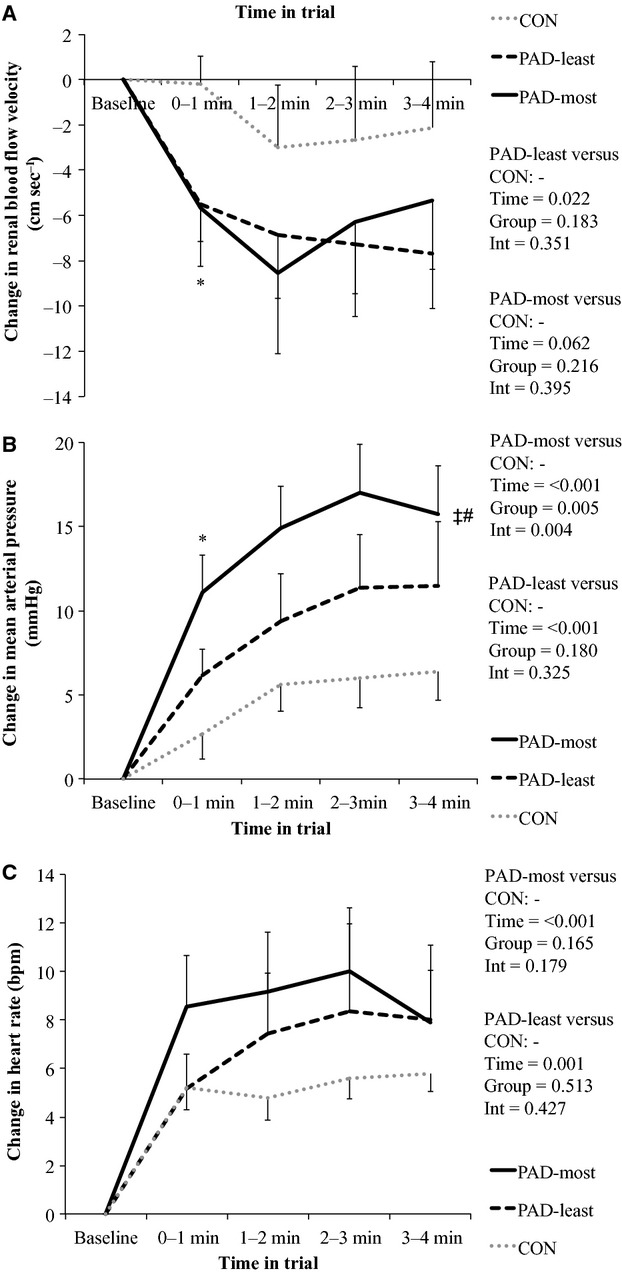
Relative change from baseline in renal blood flow velocity (A), mean arterial blood pressure (B), and heart rate (C) for peripheral arterial disease (PAD) patients and age-matched, healthy controls (CON) during 4 min of mild, rhythmic, plantar flexion exercise of increasing intensity (0.5–2 kg) with each leg (most and least affected legs for PAD; first leg for CON). Int, interaction. *Significantly different from CON at specific time point (*P* < 0.05). ^‡^Significantly different from CON (*P* < 0.05). ^#^Significantly different from CON over 4-min exercise (*P* < 0.05). Note that mean arterial blood pressure and heart rate data are similar to data published in our previous study (see Subjects section in Methods; Muller et al. 2012).

**Figure 3:**
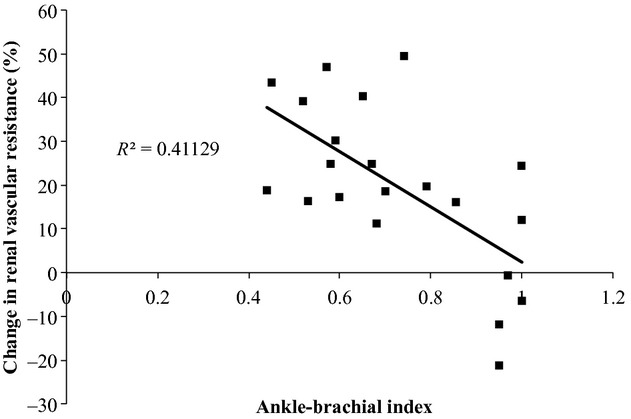
Relationship between ankle-brachial index and percent change from baseline in renal vascular resistance in peripheral arterial disease patients during the first 20 sec of mild, rhythmic, plantar flexion exercise at a 0.5-kg workload with their most and least affected legs.

Exercise significantly increased RVR, MAP, and HR and decreased RBFV from baseline (*P* < 0.05; Figs. 1, 2). PAD-most had significantly greater increases in RVR and MAP during the 4-min exercise than CON (*P* < 0.05). PAD-least also had significantly greater increases in RVR during the 4-min exercise than CON (*P* < 0.05). Within the first minute of exercise, PAD-most had significantly greater increases in RVR and MAP, and a decrease in RBFV than CON (PAD-most: 26 ± 5%, 11 ± 2 mmHg and −5.7 ± 1.5 cm sec^−1^ vs. CON: 3 ± 3%, 3 ± 2 mmHg and −0.2 ± 1.2 cm sec^−1^ for RVR, MAP, and RBFV, respectively; *P* < 0.05). PAD-least also had a significantly greater increase in RVR than CON within the first minute of exercise (PAD-least: 17 ± 5% vs. CON: 3 ± 3%; *P* < 0.05). Additionally, there was a moderate, inverse relationship between ABI and RVR change from baseline in PAD patients within the first 20 sec of exercise with their most and least affected legs (Fig. 3). RPE scores were similar in PAD-most, PAD-least, and CON during the 4-min exercise (Table 2). PAD-most had significantly higher pain scores in the third and fourth minutes of exercise than CON (*P* < 0.05; Table 2).

**Table 2:** Ratings of perceived exertion (RPE, 6–20 scale) and pain (0–10 scale) for PAD patients and age-matched, healthy controls during 4 min of mild, rhythmic, plantar flexion exercise of increasing intensity (0.5–2 kg) with each leg (most and least affected legs for PAD) and with and without AA

			0.5 kg	1 kg	1.5 kg	2 kg
RPE	PAD (*n* = 11)	Most affected leg	9 ± 1	11 ± 1	11 ± 1	11 ± 1
		Least affected leg	8 ± 1	9 ± 1	10 ± 1	10 ± 1
	CON (*n* = 10)	First leg	8 ± 0	10 ± 0	11 ± 0	11 ± 0
		Second leg	9 ± 0	9 ± 0	11 ± 0	11 ± 0
	PAD (*n* = 8)	Most affected leg	10 ± 1	11 ± 1	12 ± 1	12 ± 1
		Most affected leg with AA	9 ± 1	10 ± 1	12 ± 1	12 ± 1
Pain	PAD (*n* = 11)	Most affected leg	1 ± 1	1 ± 1	2 ± 1*	2 ± 1*
		Least affected leg	1 ± 0	1 ± 0	1 ± 1	1 ± 1
	CON (*n* = 10)	First leg	0 ± 0	0 ± 0	0 ± 0	0 ± 0
		Second leg	0 ± 0	0 ± 0	0 ± 0	0 ± 0
	PAD (*n* = 8)	Most affected leg	1 ± 1	1 ± 1	2 ± 1	2 ± 1
		Most affected leg with AA	1 ± 0	2 ± 1	2 ± 1	3 ± 1

Note that data are similar to data published in our previous study (see Subjects section in Methods; Muller et al. 2012). PAD, peripheral arterial disease; AA, ascorbic acid; CON, controls.

* Significantly different from CON (*P* < 0.05).

**Figure 4:**
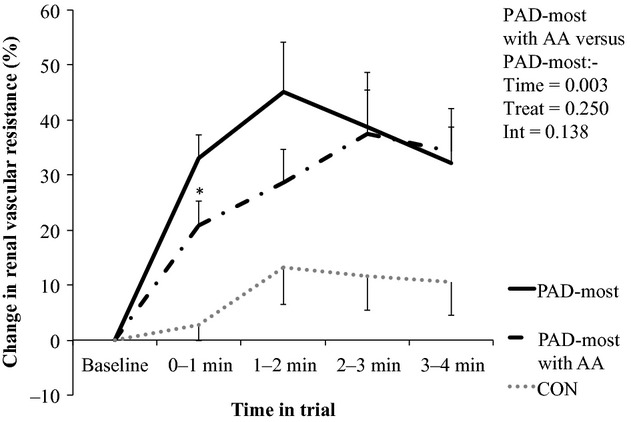
Percent change from baseline in renal vascular resistance for peripheral arterial disease (PAD) patients without and with ascorbic acid (AA) during 4 min of mild, rhythmic, plantar flexion exercise of increasing intensity (0.5–2 kg) with their most affected leg. Age-matched, healthy controls’ (CON) responses to exercise with their first leg are shown for reference. Treat, treatment; Int, interaction. *Significantly different from PAD-most at specific time point (*P* < 0.05).

**Figure 5:**
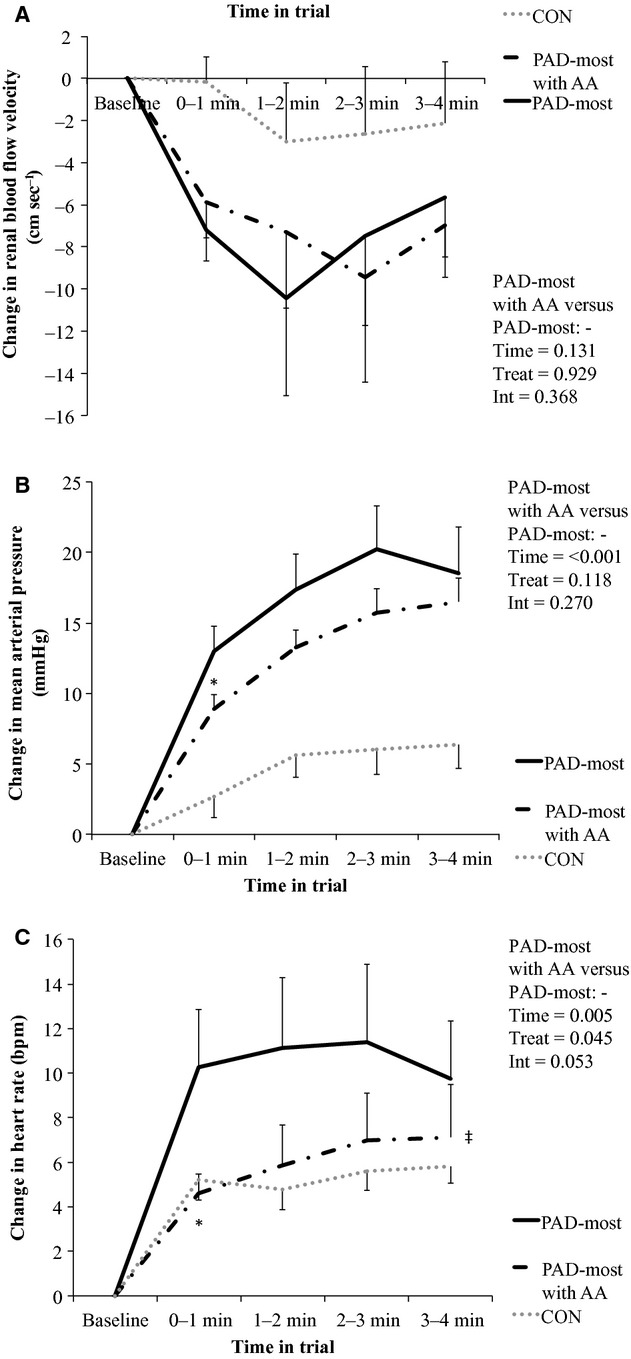
Relative change from baseline in renal blood flow velocity (A), mean arterial blood pressure (B), and heart rate (C) for peripheral arterial disease (PAD) patients without and with ascorbic acid (AA) during 4 min of mild, rhythmic, plantar flexion exercise of increasing intensity (0.5–2 kg) with their most affected leg. Age-matched, healthy controls (CON) responses to exercise with their first leg are shown for reference. Treat, treatment; Int, interaction. *Significantly different from PAD-most at specific time point (*P* < 0.05). ^‡^Significantly different from PAD-most (*P* < 0.05). Note that mean arterial blood pressure and heart rate data are similar to data published in our previous study (see Subjects section in Methods; Muller et al. 2012).

AA did not alter baseline RVR, RBFV, MAP, or HR in PAD patients (PAD-most: 2.1 ± 0.3 au, 52.4 ± 8.2cm sec^−1^, 94 ± 3 mmHg, and 73 ± 3 bpm vs. PAD-most with AA: 1.9 ± 0.3 au, 59.5 ± 8.7 cm sec^−1^, 99 ± 4mmHg, and 68 ± 3 bpm for RVR, RBFV, MAP, and HR, respectively; *n* = 8). AA did not affect the RVR and MAP increases or the RBFV decrease but did significantly attenuate the HR increase during the 4-min exercise in PAD-most (*P* < 0.05; Figs. 4, 5). Within the first minute of exercise, AA significantly attenuated the RVR, MAP, and HR increases in PAD-most (PAD-most: 33 ± 4%, 13 ± 2 mmHg, and 10 ± 3 bpm vs. PAD-most with AA: 21 ± 4%, 9 ± 1 mmHg, and 5 ± 1 bpm for RVR, MAP and HR, respectively; *P* < 0.05). AA did not affect RPE or pain scores in PAD-most during the 4-min exercise (Table 2).

## Discussion

Results from this study show that RVR was augmented during mild, one-legged, plantar flexion exercise in PAD patients compared with CON subjects. This indicates that PAD patients have augmented renal vasoconstriction during exercise. Additionally, AA infusion attenuated this augmented RVR increase within the first minute of exercise. This suggests that oxidative stress contributes toward this exaggerated response.

Augmented renal vasoconstriction occurs during exercise in heart failure patients (Middlekauff et al. 2000), with the contribution of the muscle mechanoreflex likely upregulated (Middlekauff et al. 2001). There is still controversy about the relative contributions of the muscle mechanoreflex and metaboreflex toward augmented cardiovascular responses in heart failure (Middlekauff et al. 2007; Middlekauff and Sinoway 2007a,b; Piepoli and Coats 2007). However, findings from this study indicate that renal vasoconstriction is also augmented in PAD patients. The augmented RVR increase occurred within the first minute of exercise, which supports the concept that exaggerated muscle mechanoreflex activation leads to elevated renal vasoconstriction, as this would likely be the predominant component of muscle afferent feedback to be activated at the onset of exercise. This finding is in agreement with studies using an animal model of PAD demonstrating that renal sympathetic nerve activity and MAP responses are augmented during muscle mechanoreflex activation in rats with femoral arterial occlusion (McCord et al. 2011; Lu et al. 2013).

The mechanism(s) involved in this potential upregulation of muscle mechanoreflex activity that likely augmented renal vasoconstriction during exercise in PAD remain(s) to be elucidated. It may be the case that atherosclerotic narrowing of blood vessels in patients’ lower limbs leading to reduced blood flow might initially enhance muscle metaboreceptor sensitivity but over time, these receptors may become desensitized due to overstimulation. Consequently, muscle mechanoreceptor sensitivity may increase in order to compensate for the reduced muscle afferent feedback, resulting in upregulation of the muscle mechanoreflex. This sequence of events is one current explanation for the apparent exaggerated muscle mechanoreflex activity in heart failure (Smith et al. 2006) and may also occur in PAD. It would be of interest to examine the influence of muscle metaboreflex activation on the augmented RVR increase during exercise in PAD patients, as it is currently unknown if and how this component of muscle afferent feedback is affected by PAD. Nevertheless, it remains that muscle mechanoreflex activation is likely upregulated during exercise in PAD patients as indicated by the early onset of the augmented RVR increase.

One potential mechanism involved in augmenting muscle mechanoreflex activity and consequently renal vasoconstriction during exercise in PAD is oxidative stress. PAD patients have higher markers of oxidative stress (Mueller et al. 2004; Loffredo et al. 2007) and we have previously shown that AA infusion to reduce oxidative stress attenuated the augmented MAP and HR increases during exercise in PAD patients (Muller et al. 2012). In the current study, the augmented RVR increase within the first minute of exercise was attenuated with AA. This reduction indicates that oxidative stress contributes in part to the exaggerated muscle mechanoreflex activation that appears to augment the RVR increase at the onset of exercise in PAD. Oxidative stress has previously been shown to augment the renal sympathetic nerve activity increase to brief (30 sec) static muscular contraction in rats with myocardial infarction, an animal model of heart failure (Koba et al. 2009). Our findings provide evidence in humans that oxidative stress likely plays a role in the apparent augmented muscle mechanoreflex activation that appears partly responsible for the exaggerated renal vasoconstriction during exercise in PAD.

The RVR increase from baseline within the first 20 sec of exercise in PAD was modestly related to patients’ ABIs, in that the lower the ABI, the greater the increase in RVR at the onset of exercise. This inverse correlation suggests that the severity of patients’ disease is linked with the exaggerated increase in renal vasoconstriction that occurs during a time when the muscle mechanoreflex is likely predominantly activated. This relationship indicates a link between reduced lower limb blood flow due to atherosclerotic narrowing of blood vessels and increased renal vasoconstriction caused by muscle mechanoreflex activation during exercise. It may be that as lower limb blood flow is reduced, this plays a role in increasing muscle mechanoreceptor sensitivity. Understanding how this may occur at the cellular level warrants further investigation.

The augmented RVR increase within the first minute of exercise in PAD occurred when patients performed the exercise with either their most or least affected leg. When set in the context of daily life, these exaggerated responses might occur often, for example when doing physical activity with the lower limbs for only one minute or less, possibly even within 20 sec (Fig. 3). As the weight used for the initial exercise stage in this study was only 0.5 kg, this might mean that even mild walking would likely lead to exaggerated renal vasoconstriction in PAD patients. In the short term, this heightened physiological response is appropriate to supply the cardiovascular demands of the exercise being performed. As blood flow delivery to the working muscles is likely limited by atherosclerotic obstructions within the lower limb blood vessels, blood flow to the renal vascular beds is reflexively reduced further to redirect more blood to active skeletal muscles where it is needed at that time. RVR was augmented for the duration of exercise when performed with either the most or least affected leg in PAD compared with CON in this study, suggesting that exaggerated renal vasoconstriction occurs for as long as exercise is performed. The plateau in RVR after the second minute of exercise might reflect the ability of the augmented MAP increase to provide more blood flow to the working muscles. In the long term, the cumulative strain likely imposed on the renal circulation by this heightened response during physical activity in patients might exacerbate their disease state and may further increase their cardiovascular risk. It should be noted that the supine small muscle mass exercise performed in this study is more of an experimental model of physical activity rather than upright exercise involving a large muscle mass, which would also be of interest to study.

Of note, the augmented RVR increase during exercise in PAD patients occurred before their reported level of pain was significantly higher than in CON subjects. The elevated renal vascular response occurred as early as the first minute of exercise, yet pain was not significantly higher in PAD patients compared with CON subjects until the third and fourth minutes of exercise. This suggests that the greater RVR increase during the beginning of exercise in PAD was not due to a pain response from performing the exercise.

There are several limitations in this study. Although we matched the CON group to the PAD patients by age, we did not match them by medications taken by the patients. However, patients withheld their antihypertensive medications on the morning of the study in an attempt to uncover their “more typical” PAD responses to exercise, without their responses being masked by the actions of their medications. We did not measure plasma AA or markers of oxidative stress to confirm the effectiveness of the antioxidant infusion, or perform a saline control trial. We did not specifically test the day-to-day variability in or reproducibility of the RVR, MAP, or HR responses. However, all of our analyses were changes from baseline, so individuals served as their own control during each visit. Also, our observation was that the RVR responses during exercise were fairly robust in that the patients consistently had greater responses than the controls. We cannot exclude the possibility that patients performed a greater relative intensity of exercise, or that they generated more central command that could have contributed to the augmented RVR response. However, the RPE scores were very similar between PAD and CON, so we feel that this possibility is unlikely.

In conclusion, the findings from this study reveal that PAD patients have augmented renal vasoconstriction during exercise, with oxidative stress contributing to this exaggerated response. These findings further our understanding of the physiological maladaptations that occur during exercise in PAD, which could have detrimental consequences for patients’ disease progression.
